# Data-Independent Acquisition Represents a Promising Alternative for Fast Photochemical Oxidation of Proteins (FPOP) Samples Analysis

**DOI:** 10.1021/acs.analchem.4c01084

**Published:** 2024-07-05

**Authors:** Marek Zakopcanik, Daniel Kavan, Zdenek Kukacka, Petr Novak, Dmitry S. Loginov

**Affiliations:** † Institute of Microbiology, The Czech Academy of Sciences, 14220 Prague, Czech Republic; ‡ Department of Biochemistry, Faculty of Science, Charles University, 12820 Prague, Czech Republic

## Abstract

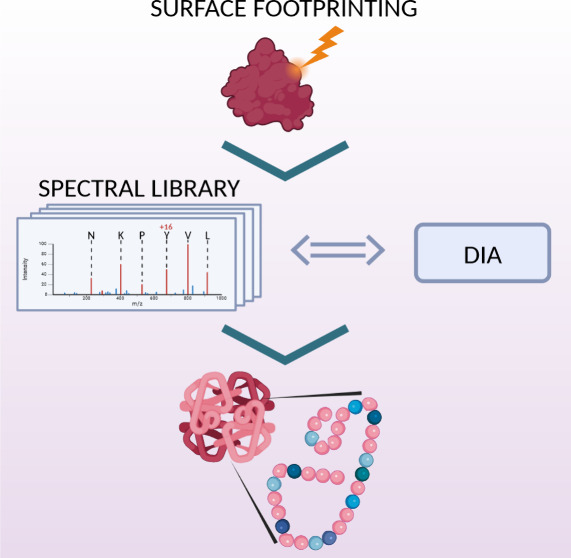

Fast Photochemical Oxidation of Proteins (FPOP) is a protein footprinting method utilizing hydroxyl radicals to provide valuable information on the solvent-accessible surface area. The extensive number of oxidative modifications that are created by FPOP is both advantageous, leading to great spatial resolution, and challenging, increasing the complexity of data processing. The precise localization of the modification together with the appropriate reproducibility is crucial to obtain relevant structural information. In this paper, we propose a novel approach combining validated spectral libraries together with utilizing DIA data. First, the DDA data searched by FragPipe are subsequently validated using Skyline software to form a spectral library. This library is then matched against the DIA data to filter out nonrepresentative IDs. In comparison with FPOP data processing using only a search engine followed by generally applied filtration steps, the manually validated spectral library offers higher confidence in identifications and increased spatial resolution. Furthermore, the reproducibility of quantification was compared for DIA, DDA, and MS-only acquisition modes on timsTOF SCP. Comparison of coefficients of variation (CV) showed that the DIA and MS acquisition modes exhibit significantly better reproducibility in quantification (CV medians 0.1233 and 0.1494, respectively) compared to the DDA mode (CV median 0.2104).

## Introduction

Nowadays, a comprehensive structural study of proteins requires not only a snapshot of one state but also dynamic information which can provide some insight into various peculiarities of protein complex formation,^1−3^ protein–DNA interactions,^4,5^ protein folding,^6,7^ etc. From this point of view, radical footprinting methods represent a valuable tool.

Protein surface footprinting provides information related to the surface accessible area of residue side chains, resulting in biologically relevant data concerning interaction or folding dynamics. Footprinting might be implemented by means of different radical probes.^8−10^ Among them, the Fast Photochemical Oxidation of Proteins (FPOP) introduced in 2005^11^ is the most popular. It is a covalent labeling-based method that utilizes hydroxyl radicals. Due to ·OH properties and their wide reactivity toward amino acids, this method has the potential to provide valuable information even on minor conformational changes of protein residue side chains.^12^ On the other hand, broad reactivity results in an extreme number of variable modifications, which should be accounted for during data processing. The precise localization of the oxidative modification in the peptide sequence is a keystone of FPOP analysis. Thus, data processing is a bottleneck in FPOP analysis. One way to overcome this issue is to restrict the spatial resolution of the method by reducing the number of modifications allowed per search.^13^

The challenge of FPOP data processing was addressed in our recent study.^14^ We showed that the results of the FPOP experiment are highly dependent on a selected peptide identification framework, with only 30% of the identified modifications mutual for the Mascot and PEAKS search engines. Applied statistical analysis in combination with manual validation of assigned modifications revealed that Mascot provided more reliable FPOP data over PEAKS. This approach allows us to reduce the time for manual validation of assigned FPOP modifications but is still quite laborious.

One of the possibilities to avoid the need for repeated ID validation for each experiment on the same set of proteins might be implementation of a reference spectral library. Linking the peptide parameters (sequence, *m*/*z*, charge, retention time, and possibly ion mobility) together with its characteristic fragmentation pattern is the underlying principle of spectral libraries.^15^ Compared to the older approach of database search, spectral library search is limited to identifications already present in the library. On the other hand, spectral library search offers greater sensitivity, is less time-consuming, provides a higher number of peptide-spectrum matches at the same error rate, and is capable of detecting less obvious matches.^16,17^ The employment of proficient control mechanisms can result in a library consisting of highly confident identifications and precisely assigned PTMs, or oxidative modifications in the case of FPOP analysis. Additionally, the implementation of library search in the FPOP data processing workflow allows the utilization of data-independent acquisition (DIA).

Data collection in mass spectrometry-based proteomics can take place in either full-scan mode or tandem MS, further divided into data-dependent acquisition (DDA) and DIA. In DDA mode, a limited number of precursor ions (topN) are selected from survey scans based on their parameters (typically the intensity and charge state) and then subjected to fragmentation, generating fragmentation spectra. In DIA, the mass spectrometer sequentially isolates and fragments predefined precursor windows across the desired mass range. This approach allows for a comprehensive coverage of precursors across various abundance levels in the defined mass range. DIA acquisition provides data with significantly less missing values, resulting in better reproducibility of the analysis over DDA mode.

Due to the extensive number of modifications that need to be considered during the evaluation of FPOP data, the formation of spectral libraries from DIA data is not yet viable. To obtain the spectral library for the HbHp complex, we opted for the combination of FragPipe for the search of the DDA data and Skyline for the validation part. The FragPipe^18^ is an open-source pipeline that offers fast and precise identification of proteomic data. Skyline^19^ is an open-source application for targeted proteomics. The software offers to generate a spectral library from FragPipe search results and view annotated fragmentation spectra associated with individual library entries. Together with dot product scoring and the option to filter on the basis of the number of matched ions, this allows for a convenient way to validate each identification. For FPOP to bear relevant structural information, the certainty of the modification position is crucial. Thus, only spectra with sufficient fragmentation coverage should be included in the spectral library.

## Methods

### Chemicals and Materials

Human haptoglobin phenotype 1–1 (Hp), human hemoglobin (Hb), and catalase were purchased from Sigma-Aldrich. PNgase F was from New England BioLabs Inc. Mass spectrometry-grade trypsin was from Promega Corporation. All solvents were purchased from Merck (Germany) in LC-MS grade. Other chemicals reported in this article were purchased from Sigma-Aldrich.

### FPOP of the HbHp Complex

Protein solutions at 0.3 mg/mL concentrations were prepared in 150 mM ammonium acetate (pH 7.0), Hp and Hb were mixed in an equimolar ratio to form a complex. FPOP analysis was carried out as described previously^2^ with minor modifications. Prior to FPOP analysis, histidine was added to the samples at 1 mM concentration. The protein complex was mixed with 20 mM H_2_O_2_ using a continuous capillary flow system (1:1 v/v) and irradiated with a laser shot (15 Hz, 20 ns pulse duration, 2.24 mJ/mm^2^ radiant exposure) by a 248 nm KrF laser (Coherent, COMPex50). Quenching of hydroxyl radicals was done with 75 mM l-methionine immediately after irradiation. The samples were collected in a plastic tube with catalase to remove H_2_O_2_ excess. The FPOP experiment was conducted in pentaplicate.

### FFAP of the HbHp Complex

The FFAP experiment was performed as described previously.^8^ Briefly, lyophilized Hp and Hb were dissolved in 50 mM ammonium carbonate buffer pH 7.4 and mixed together in 1:1 ratio. The equimolar mixture of Hp and Hb (0.3 mg/mL) was labeled by acetic Togni reagent dissolved in dimethylsulfoxide (50 mg/mL) upon activation by l-Ascorbic acid (10 mg/mL). The final concentration of acetic Togni reagent in the reaction was 7.5 mM. The labeling reaction was stopped after 3 s by adding tryptophan (10 mg/mL). Five μg of the labeled complex was desalted using a C4 microtrap column (Optimize Technologies, Oregon City, OR) and dried using a SpeedVac concentrator. Experiment was done in biological triplicate.

### Protein Digestion and LC-MS and MS/MS Data Acquisition

The collected FPOP samples were diluted (3:1 ratio) with 150 mM 4-ethylmorpholine buffer (pH 8.5), supplemented with 15% acetonitrile (ACN), and FFAP samples were dissolved in 50 mM 4-ethylmorpholine buffer (pH 8.5), supplemented with 5% ACN. Reduction and alkylation were achieved by adding tris(2-carboxyethyl)phosphine and 2-chloroacetamide at 10 mM and 30 mM concentrations, respectively. The samples were incubated at 70 °C for 5 min and cooled down on a bench. Deglycosylation was carried out by adding PNGase F in 1:20 ratio followed by overnight incubation at 37 °C. Subsequently, trypsin was added to the samples in a 1:20 ratio and the samples were digested for 8 h at 37 °C. The digestion was terminated by adding trifluoroacetic acid (TFA) to a final concentration of 0.2%. Samples were desalted using a C18 microtrap column (Optimize Technologies, Oregon City, OR) and dried using a SpeedVac concentrator.

The samples were resuspended in 20 μL of 2% ACN, 0.1% TFA. In addition, a pooled sample was prepared by mixing an equal volume of all replicates in triplicate. All samples were analyzed using the Vanquish Neo UHPLC liquid chromatography system (Thermo Scientific) coupled with a timsTOF SCP mass spectrometer equipped with Captive spray (Bruker Daltonics). LC conditions were the same for all acquisition modes (DDA, MS1, and DIA). The peptides were trapped in the C18 trap column (PepMap Neo C18 5 μm, 300 μm × 5 mm, Thermo Scientific). After 3 min of trapping, the peptides were eluted from the trap column and separated on a C18 column (DNV PepMap Neo 75 μm × 150 mm, 2 μm, Thermo Scientific) by a 55 min long linear 0.1% formic acid (FA)–ACN gradient from 5% (v/v) to 35% (v/v) ACN at a flow rate of 350 nL/min. Both columns were heated to 50 °C. The DDA acquisition was done using parameters from the standard proteomic PASEF method. Spectra were recorded within the range of 100–1700 *m*/*z*, while the ion mobility was scanned from 0.7 to 1.3 Vs/cm^2^. The method consisted of a TIMS survey scan lasting 166 ms, followed by 5 PASEF MS/MS scans. The total cycle time for this process was 1.04 s. The target intensity was set at 14000 with an intensity threshold of 500. The precursors for data-dependent acquisition were fragmented with an ion mobility-dependent collision energy that increased linearly from 20 to 59 eV. Active exclusion was enabled for 0.4 min with the mass width 0.015 *m*/*z* and 1/K0 width 0.015 V × s/cm^2^ to prevent repeated selection of the same precursor ions. The same method with switched off PASEF mode was used to collect only MS1 scans.

DIA acquisition was done using the standard dia-PASEF method. Spectra were recorded within the range of 100–1700 *m*/*z*, while the ion mobility was scanned from 0.64 to 1.45 Vs/cm^2^. The number of dia-PASEF scans was 24, separated into three ion mobility windows per scan covering an *m*/*z* range from 400 to 1000 by 25 Th windows and an ion mobility range from 0.64 to 1.37 Vs/cm^2^ with an estimated cycle time of 0.96 s. The accumulation and the ramp time was 100 ms, and collision energy settings were the same as in the DDA mode. The mass spectrometry proteomics data have been deposited to the ProteomeXchange Consortium^20^ via the PRIDE^21^ partner repository with the data set identifier PXD050132 and DOI 10.6019/PXD050132.

### Data Analysis

Data obtained in DDA mode were searched using FragPipe 20.0 (Nesvizhskii Lab, University of Michigan, MI, USA against a database containing sequences of Hb (Uniprot IDs P69905 and P68871) and Hp (Uniprot ID P00738) supplemented with a deglycosylated form of β subunit, together with 246 sequences of common contaminants (including trypsin, PNGase F, and catalase). For each database entry, a reverse decoy sequence was appended. The precursor ion tolerance was set to 12 ppm, and the fragment mass tolerance was set to 0.05 Da. Only tryptic peptides (without cleavage before P) with up to two misscleavages were considered. Cysteine carboxymethylation was defined as fixed modification and FPOP modifications were denoted as variable modifications (Table S1). A maximum of 2 variable modifications per peptide was allowed. This limitation was chosen in order to operate within reasonable search space. On behalf of validation, the MSBooster rescoring was employed together with Percolator (version 3.06) and ProteinProphet (part of Philosopher 5.0).

Data collected from pooled samples was processed using Mascot as described in Zakopcanik et al.^14^ Briefly, raw data were converted to MGF files in Compass DataAnalysis v.5.3 (Bruker Daltonics, Billerice, MA, USA). The files obtained were then searched using Mascot 2.7.0 (Matrix Science Inc., Boston, MA, USA). The search parameters were set the same as for FragPipe search (12 ppm precursor tolerance, 0.05 Da fragment tolerance, same cleavage rules and same database). Because the number of variable modifications being limited in Mascot, trioxidation was not included. The significance threshold was set at *p* ≤ 0.05. Only identifications present in all replicates were accepted.

The results of the FragPipe search were processed in Skyline 23.1.0.268 (MacCoss Lab, University of Washington, Seattle, WA, USA). First, the five *interact.pep.xml* files were loaded together with five uncalibrated *mgf* files (generated by FragPipe). The cutoff score calculated in the *filter.log* file was not used, since the FPOP results represent a heterogeneous data set and would require separate thresholds for each group of identifications.^22^ The precursor charges were set to 2–4 with a mass accuracy of 12 ppm. Fragment ions were restricted to *b* and *y*, with charges to 1 and 2 and library ion match tolerance of 0.05 *m*/*z*. The ion mobility resolving power was kept at the default value of 30. Trypsin [KR|P] with a maximum of 2 misscleavages was set for the digestion of the Hb and Hp sequences and up to 2 FPOP modifications, defined the same way as for FragPipe search. Each entry of the resulting library was manually inspected. Several aspects were considered during the validation. First, the overall sequence fragmentation coverage, while ensuring that the annotated fragments represent the majority of the tandem spectrum. Second, the Skyline-calculated dot product score, comparing the theoretical profile of the library entry and the actual isotopic distribution of the precursor in each of the replicates. All IDs that scored less than 0.9 of the isotopic dot product were closely inspected, which helped to detect misassignments, mainly carbonyl IDs that were wrongly assigned to peptides modified with single oxidation. Finally, special attention was paid to the presence of fragment ions that determine the exact position of the modification. In case that the modification could not be precisely localized, it was included as an “unlocalized mass shift”. Identifications with low precursor intensity (below 2500 in Skyline notation) were excluded from the library. As a next step of validation, the spectral library was used as a reference to match against the DIA data in Skyline. A dot product score was used as a measure of spectral similarity. All spectra with a dot product score less than 0.7 were selected for closer examination.

Next, DIA and MS-only acquired data were mapped onto a respective library match using *m*/*z*, RT, and dot product score as criteria in Skyline software. Confident identifications were quantified in each of the acquisition modes at MS1 level using Compass DataAnalysis 5.3 (Bruker Daltonics, Billerice, MA, USA). Based on our previous experience with FPOP data, identifications were quantified in charge states 1–5, which is necessary to encompass complete information about the extent of modification. The extent of modification (EoM) itself was calculated for each identification individually as the intensity of the modified peptide divided by the sum of intensities of the unmodified peptide and all of its modified variants. The proportion of unmodified peptide was calculated using the same formula.
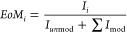


## Results and Discussion

**Figure 1:**
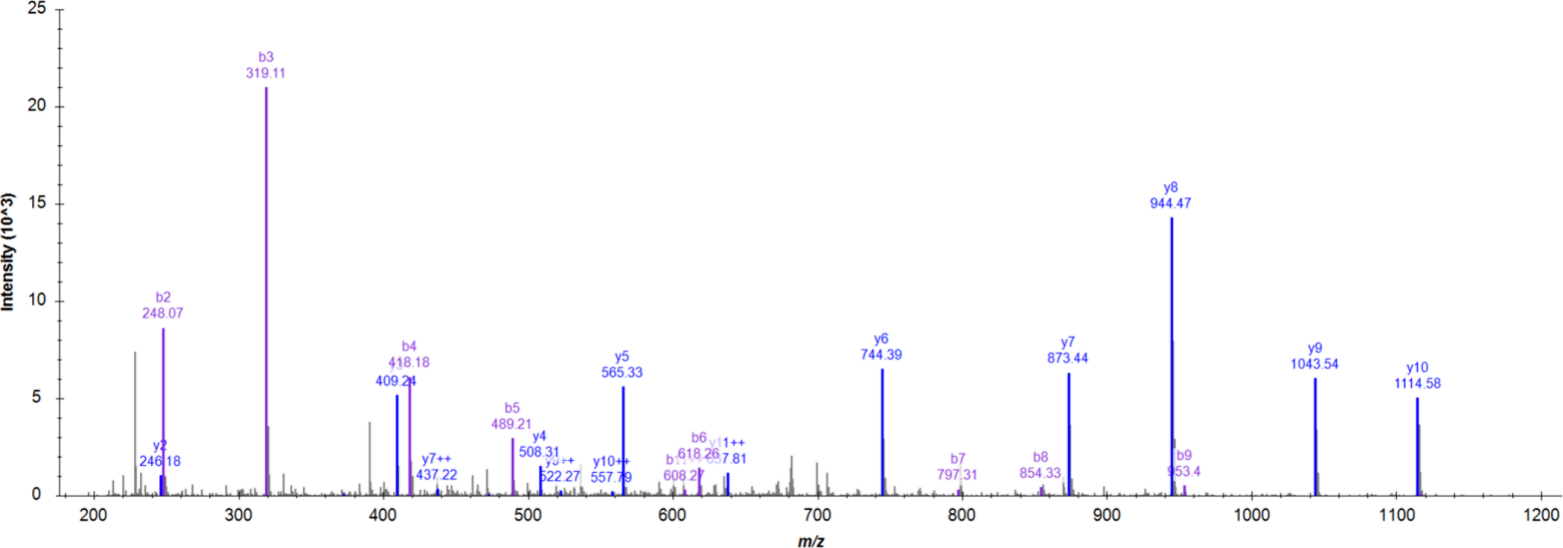
Fragmentation spectrum of Tyr7 oxidation in the peptide SCAVAEYGVYVK in its doubly charged form at *m*/*z* 681.32 and RT 16.70 min. The spectrum contains 19 out of 22 possible fragments, with y5 and y6 providing confidence for the modification site, representing optimal entry for the spectral library.

**Figure 2:**
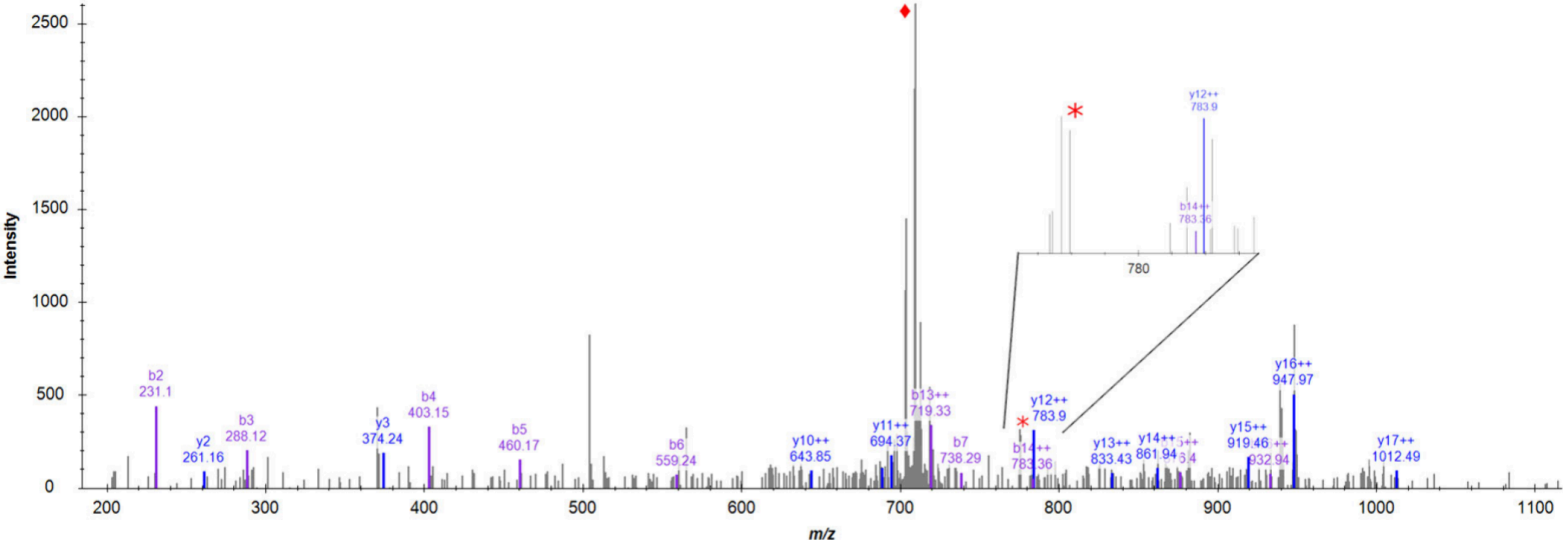
Fragmentation spectrum of Tyr7 oxidation in a peptide TEGDGVYTLNNEKQWINK in its triply charged form at *m*/*z* 709.01 and RT 12.22 min. Most of the assigned fragment ions have low intensity. Additionally, the doubly charged y12 fragment, which should support the localization of the modification, has an unmodified counterpart with the same intensity (labeled *). Analysis of raw data revealed at least two coeluted peptide species separated by ion mobility. The ⧫ symbol denotes the precursor. Because of the uncertainty of the modification site, together with the low intensity of the assigned fragment ions, this ID was excluded from the spectral library.

The optimization of the DIA workflow was performed on the FPOP oxidized HbHp protein complex, which represents a complex protein sample containing disulfide bonds and carbohydrate part. The HbHp complex was measured in pentaplicates on timsTOF SCP in two MS/MS acquisition modes—DDA, DIA—and once in MS-only mode. The DDA analyses were searched using FragPipe, and the results were loaded into Skyline to form a spectral library. The parameters considered during manual validation were the isotopic profile of the precursor peptide, the dot product of the fragment ion spectra, the number of assigned fragment ions, and the fragmentation coverage of the site of modification. For example, the fragmentation spectrum of the peptide SCAVAEYGVYVK at *m*/*z* 681.32 (doubly charged) and RT 16.70 min bearing oxidation on Tyr7 (Figure 1) shows almost complete fragmentation coverage (19 out of 22 possible fragments). Furthermore, the site of modification is unambiguously determined by fragments y5 and y6. Together with the average dot product score of 0.96, this spectrum represents the ideal library entry. On the other hand, the fragmentation spectrum of the peptide TEGDGVYTLNNEKQWINK at *m*/*z* 709.01 (triply charged) and RT 12.22 min bearing oxidation on Tyr7 (Figure 2) consists of 21 fragments (of 36 possible), many of them at a borderline noise level. The modification site was assigned based on the y12^+2^ fragment, but the spectrum contains a contradictory signal of the unmodified y12^+2^ ion, indicating the possible presence of other coeluted peptides. This was further supported by examining the ion mobility information where the presence of two forms was revealed (Figure S1). Since the modification site cannot be determined confidently, the intensity of the assigned fragments is low, and the dot product score is low (0.62), this identification was rejected from the spectral library. Of 1780 identified peptides by the FragPipe search, a total of 522 were accepted, of which 447 bear the FPOP modification. This validation step produced a spectral library that included the retention time of the identifications. Although manual validation is time-consuming, the created spectral library can now be used for all future analyses of the same set of proteins (when employing the same chromatography conditions).

**Figure 3:**
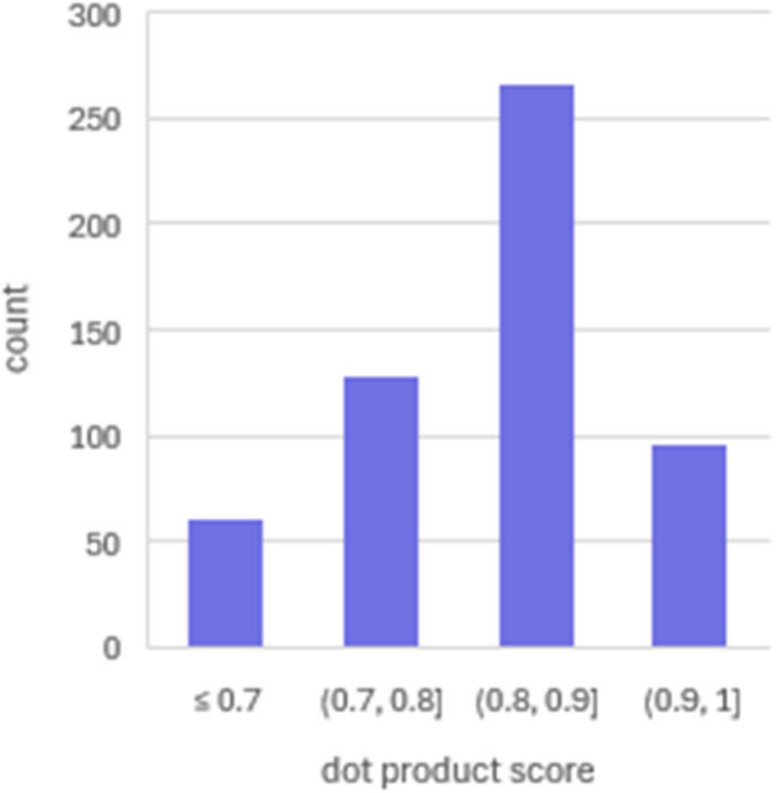
Distribution of the dot product score for DIA data matched against the manually validated spectral library.

Next, the created spectral library was matched against the DIA data to calculate the dot product score (Figure 3). Most IDs exhibit a dot product score greater than 0.8, indicating high similarity between the spectra. Identifications with a dot product score lower than 0.7 were selected for closer examination. The low dot product score could be an indication that the modification does not occur in all replicates or is of very low abundance. Therefore, even if the identification comes from a good quality fragmentation spectrum, the modified species is not representative of the state of the protein. Using raw data for validation of these low-scoring modified peptides, an additional 28 IDs were excluded from the data set. The step of matching the identifications against DIA data represents an important additional layer of validation.

The identifications obtained by this novel approach were compared with a routine workflow employing Mascot and automated filtering steps, as described previously.^14^ Due to the nature of the previously published approach, only IDs with a single FPOP modification and a precise localization of the modification site were used for this comparison. The set of manually validated modifications consists mainly of oxidations (60%), with dioxidations being the second most common modification (11%), followed by carbonylation, decarboxylation, and His +5 Da (each 7%). The complete overview of the modifications is available in Table S2. The ratio of the types of modifications is similar to the results obtained by employing the Mascot search engine, but the number of modified IDs from Mascot search (256) is lower than in the manually validated library (333). To put this comparison in more context, the following aspects of each approach should be considered. Apart from utilizing information from five replicates in the case of library approach, versus three pooled replicates in the case of the Mascot search approach, the main difference lies in the validation mechanism. The approach using Mascot represents automated filtering in the form of accepting only IDs which were detected in all of the replicates. On the other hand, for the construction of the manually validated library, only one identification from the FragPipe database search was sufficient (in the case of no conflicting IDs from other replicates). Although manual validation of the data set requires more time, once it is finished, the library can be used repeatedly for every future experiment on this set of proteins. Furthermore, it is worth noting that the Mascot results contain IDs of isobaric peptides from different retention times as separate entries. When comparing the number of IDs without isobaric forms, the difference becomes even more apparent (library: 333 IDs, Mascot: 200 IDs). The set of additional IDs present in the manually validated library included modifications of less reactive aliphatic amino acids (Val, Leu, Pro) as well as more reactive ones (His, Met, Phe, Trp, and Tyr). Several of the IDs present exclusively in the manually validated data set were from the interaction surface of the Hb and Hp subunits of the complex. This demonstrates that the additional effort results in better coverage globally, as well as in the biologically relevant areas of the protein complex. Taken together, the approach using manual validation of the identifications leads to a wider population of unique modifications and increased spatial resolution, compared to routine filtering steps, requiring the ID to be present in all replicates as a validation mechanism. However, the lack of isobaric peptides could result in an incomplete assessment of the extent of modification in the case of aromatic amino acids.^23,24^ To incorporate isobaric peptides into the proposed workflow, manual intervention is required during the validation step using Skyline. The user interface allows multiple entries of the same peptide (and the same modification) with different retention times. Such occurrences could be discovered using the extracted ion chromatogram, which Skyline reconstructs from the peak list data. DDA or DIA data would be needed for further confirmation of such IDs.

**Figure 4:**
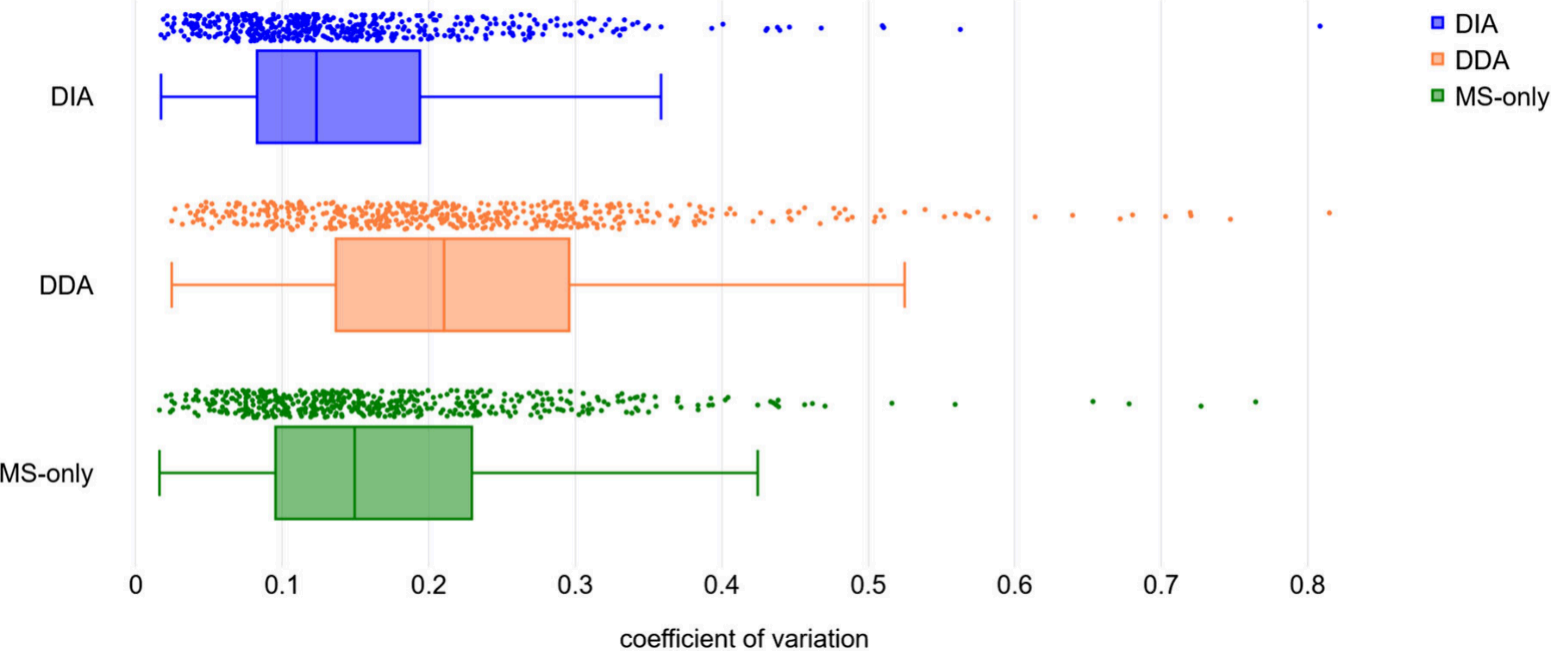
Variability of quantification. Coefficients of variation were calculated from the quantification of identified modifications (five replicates) for each acquisition mode. The line represents the median. Created with box and whisker plot maker.^25^

To validate the novel approach of using the DIA acquisition for FPOP, we compared the quantification of the accepted identifications between DIA, DDA, and MS-only modes. For each ID, the extent of modification was calculated. In order to evaluate the reproducibility of each acquisition method, a coefficient of variation (CV) was determined for each identification. Figure 4 shows the distribution of CVs for DIA, DDA, and MS-only using boxplots. More detailed parameters of the CV distribution are shown in Table 1. The DIA acquisition mode exhibits the lowest median (0.1233), as well as the lowest interquartile range, indicating the lowest variability. The characteristics of the MS-only data set are close to those of the DIA data set, with a slightly higher median (0.1494) and a broader interquartile range. However, the DDA data set varies the most, with the median of 0.2104 and the widest interquartile range of the three acquisition modes. The extents of modification of selected peptides representing low, medium, and high intensity species are plotted in the Figure S2. These results suggest that the DIA mode, together with the MS-only mode, brings better reproducibility in the quantification of the FPOP data.

**Table 1:** Parameters of CVs for DIA, DDA, and MS-Only Acquisition Methods

	**DIA**	**DDA**	**MS-only**
**Minimum:**	0.0172	0.0245	0.0161
**Q1:**	0.0828	0.1366	0.0954
**Median:**	0.1233	0.2104	0.1494
**Q3:**	0.1939	0.2958	0.2294
**Maximum:**	0.8085	0.8148	0.7645
**Mean (x̅):**	0.1501	0.2301	0.1748

To assess whether there are significant differences in variability (relative to means) among the three data sets, a one-way ANOVA test was performed.^26^ The ratio of variation between the sample means and the variation within the samples (F-statistic) is 58.067, which exceeds the threshold of H_0_ acceptance threshold (3.0024). This, together with the *p*-value converging to 0, indicates that there is a significant difference in variability between at least two of the data sets. Posthoc analysis using the Tukey Honestly Significant Difference test showed that the differences between all of the test pairs are significant (*p* < 0.01). While the CV values for the DDA mode differ substantially, with the differences of the DIA-DDA and DDA-MS-only pairs with a *p*-value lower than 1.8 × 10^–10^, the *p*-value of the DIA-MS-only pair is equal to 0.00338. The complete results for the ANOVA and the Tukey Honestly Significant Difference test are available in Tables S3 and S4.

**Figure 5:**
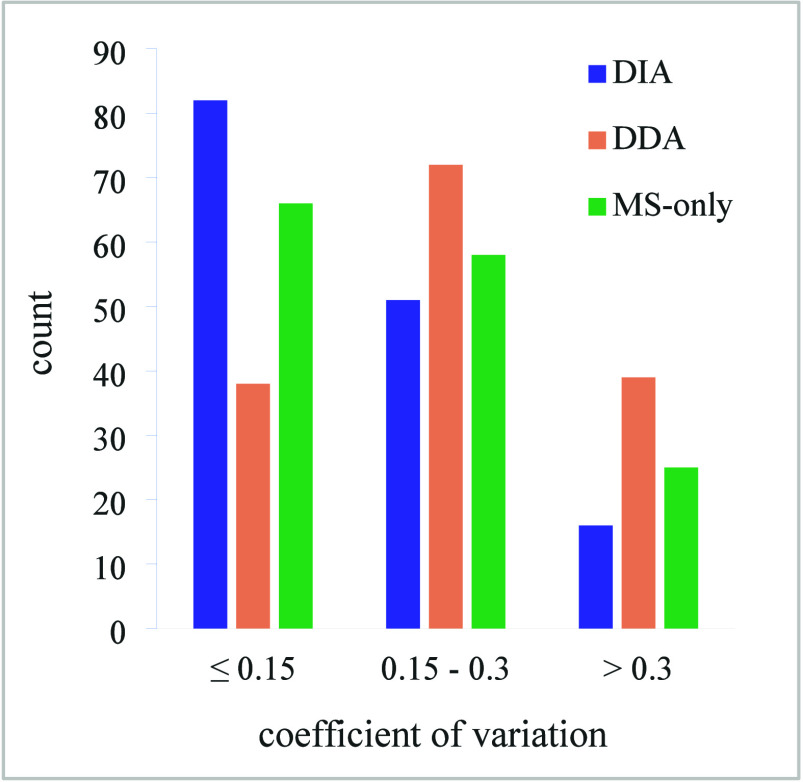
Coefficients of variation for the lowest-intensity tertile of identifications.

Furthermore, low-intensity identifications were inspected separately, as a suspected source of variability. The distribution of the lowest intensity tertile for each acquisition mode (Figure 5) retains the similarity between the DIA and MS modes. Compared to DDA, quantification from DIA or MS-only data offers higher reproducibility even for low-intensity identifications.

The proposed workflow is a universal approach that can be very effective regardless of the radical probes used for protein footprinting. Fast fluoroalkylation of proteins (FFAP) was selected as an alternative labeling technique to demonstrate the effectivity of the DIA workflow. FFAP utilizes fluoroalkyl radicals, generated by the reductive decomposition of Togni reagents with ascorbic acid, to label proteins within seconds at aromatic residues.^8^ The manually validated FFAP spectral library of the HbHp complex contained 74 Togni modified identifications. The mapping of the DIA data hinted at 15 IDs with the dot product score ≤0.7 (Figure S3), and 11 of them were removed from the validated library due to either low precursor intensity or nonconsistent presence in the replicates. Overall, the FFAP data set of the HbHp complex contained 57 reliable modifications after removing isobaric and low intensive peptides (after quantification in DataAnalysis). The extent of modification and coefficients of variation were calculated in the same way as those for the FPOP analysis. Similarly to FPOP results, quantification of FFAP data acquired in DIA mode has the lowest variability compared to DDA and MS-only acquisition modes (Figure S4). DIA data exhibit the lowest median variability (0.1382) compared to DDA and MS-only, with medians 0.1689 and 0.1781, respectively (Table S5).

## Conclusions

In this paper, we have demonstrated the potential of DIA for the protein footprinting data evaluation. The proposed workflow combines DDA data to create a validated spectral library, which is then matched against DIA data for further improvement of the IDs reliability.

Furthermore, the reproducibility of DIA, DDA, and MS-only acquisition modes for FPOP and FFAP analysis on timsTOF SCP was compared. The variability of quantification was assessed by coefficients of variation. The results reported here show that quantification from DIA data offers significantly better reproducibility compared to DDA and MS-only data.

The proposed workflow would undoubtedly benefit from automation, specifically in the step of validation of the identified spectra. Such automation could include evaluation of the fragmentation coverage of the modified peptide, and/or a carefully selected intensity threshold to cut off collision spectra originating from low abundant precursors, which often lead to spectra of insufficient quality.

## Supporting Information Available

Overview of defined variable modifications (Table S1); Extracted ion mobilogram of the peptide TEGDGVYTLNNEKQWINK (Figure S1); Representation of modifications in the validated spectral library and the Mascot search results (Table S2); Extent of modifications of selected peptides (Figure S2); Results of one-way ANOVA test (Table S3); Results of Tukey Honestly Significant Difference test (Table S4); Dot product score for DIA data matched against the spectral library for the Togni labeled data set (Figure S3); Variability of quantification of FFAP experiment (Figure S4); CV distribution of quantification for FFAP experiment (Table S5). (PDF)

## Abbreviations

ACN: acetonitrile
CV: coefficient of variation
DDA: data-dependent acquisition
DIA: data-independent acquisition
FA: formic acid
FFAP: Fast Fluoroalkylation of Proteins
FPOP: Fast Photochemical Oxidation of Proteins
Hb: hemoglobin
Hp: haptoglobin
HbHp: hemoglobin–haptoglobin
PSM: peptide-spectrum match
RT: retention time (min)
TFA: trifluoroacetic acid
