# Supplementary material for: Data-Independent Acquisition Represents a Promising Alternative for Fast Photochemical Oxidation of Proteins (FPOP) Samples Analysis
Source: Anal Chem. 2024 Jul 5;96(28):11273–9. doi: 10.1021/acs.analchem.4c01084 (PMC11256011; doi:10.1021/acs.analchem.4c01084)
Supplement: Supplementary file 1 — ac4c01084_si_001.pdf [file ac4c01084_si_001.pdf]

SUPPLEMENTARY DATA

Data-independent acquisition represents a promising alternative for FPOP samples analysis

Marek Zakopcanik[1,2], Daniel Kavan[1], Zdenek Kukacka[1], Petr Novak[1], Dmitry S. Loginov[*,1]

[1]Institute of Microbiology, The Czech Academy of Sciences, 14220 Prague, Czech Republic

[2]Faculty of Science, Charles University, 12843 Prague, Czech Republic

*Email: dmitry.loginov@biomed.cas.cz

List of Supplementary data

Overview of defined variable modifications (Table S1); Extracted ion mobilogram of the peptide TEGDGVYTLNNEKQWINK (Figure S1); Representation of modifications in the validated spectral library and Mascot search results (Table S2); Extent of modifications of selected peptides (Figure S2); Results of one-way ANOVA test (Table S3); Results of Tukey Honestly Significant Difference test (Table S4); Dot product score for DIA data matched against the spectral library for the Togni labeled data set (Figure S3); Variability of quantification of FFAP experiment (Figure S4); CV distribution of quantification for FFAP experiment (Table S5).

Table S1. Overview of defined variable modifications.

modification name	mass difference (Da)	amino acid
FPOP oxidation	+15.994915	F, H, I, K, L, M, P, R, V, W, Y
FPOP dioxidation	+31.989828	F, M, W, Y
FPOP carbonyl	+13.979265	E, H, I, L, P, Q, R, V
FPOP decarboxylation	-30.010565	E, D
FPOP Met-32Da	-32.008457	M
FPOP His+5Da	+4.9789	H
FPOP His-10Da	-10.031968	H
FPOP Arg deguanidination	-43.053432	R
FPOP trioxidation*	+47.984745	W

* not defined for Mascot

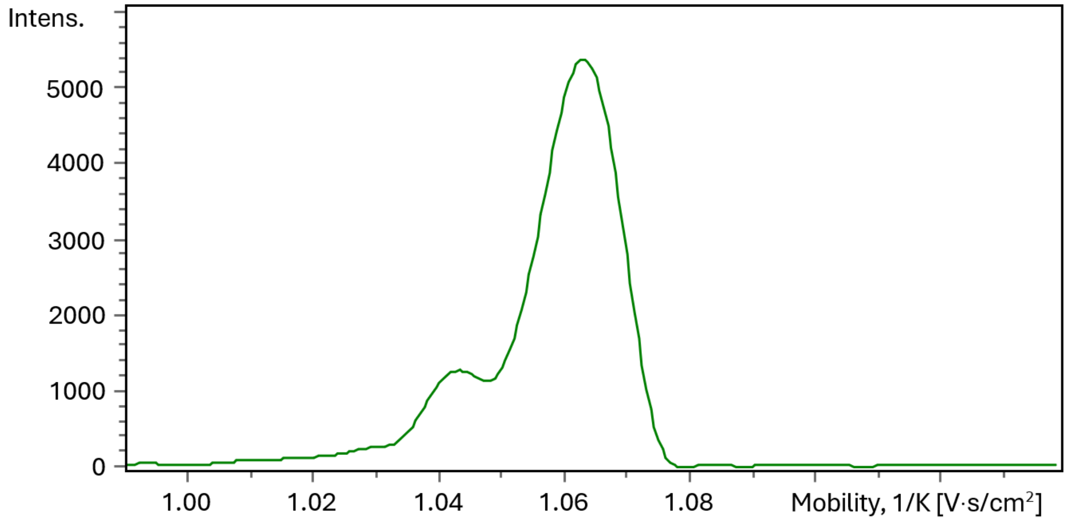

Figure S1. Extracted ion mobilogram of the peptide TEGDGVYTLNNEKQWINK at m/z 709.01 (triply charged) and RT 12.22 min bearing oxidation presumably on Tyr7. At least two coeluted subspecies separated by ion mobility explain the ambiguity of the localization of the oxidation from the MS/MS spectrum.

Table S2. Representation of modifications in the validated spectral library and in the Mascot search results.

modification	validated library		Mascot*	
	count	percentage	count	percentage
Arg deguanidination	4	1%	2	1%
carbonyl	24	7%	20	10%
decarboxylation	23	7%	18	9%
dioxidation	36	11%	17	9%
His +5Da	23	7%	10	5%
His -10Da	16	5%	9	5%
Met -32Da	5	2%	3	2%
oxidation	200	60%	121	61%
trioxidation	2	1%	N/A	N/A

* only unique modified peptides

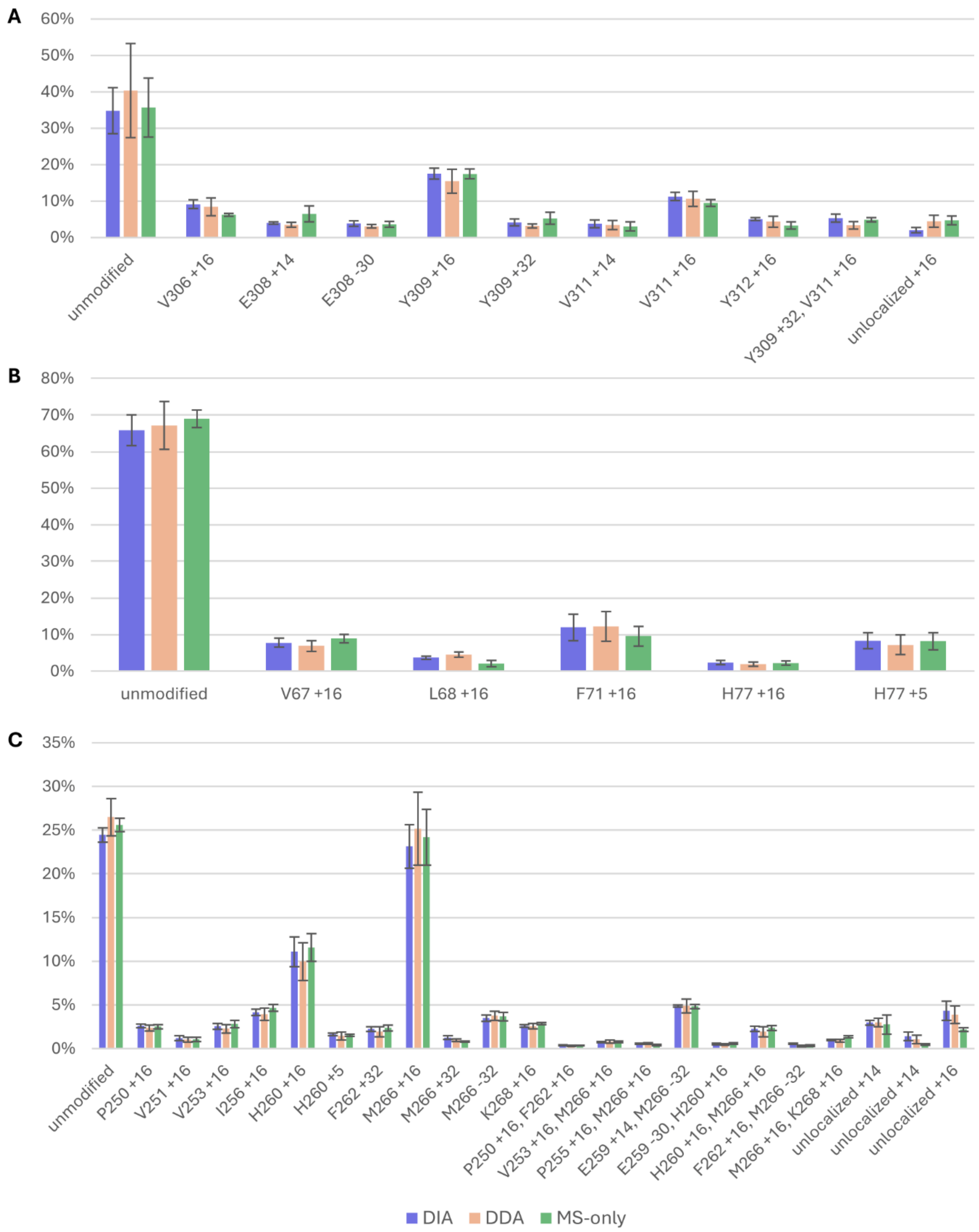

Figure S2. The extents of modification and the unmodified fraction of selected peptides. (A) Low intensity peptide SCAVAEYGVYVK; (B) medium intensity peptide KVLGAFSDGLAHLDNLK; (C) high intensity peptide SPVGVQPILNEHTFCAGMSK. The standard deviation shows that the DDA data exhibit greater variability over the DIA and MS data.

Table S3. Results of one-way ANOVA test for CVs of quantification from DIA, DDA and MS acquisition modes.

Source	DF	Sum of Square	Mean Square	F Statistic	P-value
**Groups (between groups)**	2	1.5001	0.75	58.0667	0
**Error (within groups)**	1338	17.2827	0.01292		
**Total**	1340	18.7827	0.01402		

Table S4. Results of Tukey Honestly Significant Difference test for CVs of quantifications from DIA, DDA and MS acquisition modes.

Pair	Difference	SE	Q	Lower CI	Upper CI	Critical Mean	p-value
**DIA-DDA**	0.08	0.005376	14.8824	0.06216	0.09784	0.01784	1.732e-10
**DIA-MS**	0.0247	0.005376	4.5975	0.00688	0.04255	0.01784	0.003377
**DDA-MS**	0.0553	0.005376	10.2849	0.03745	0.07312	0.01784	1.75e-10

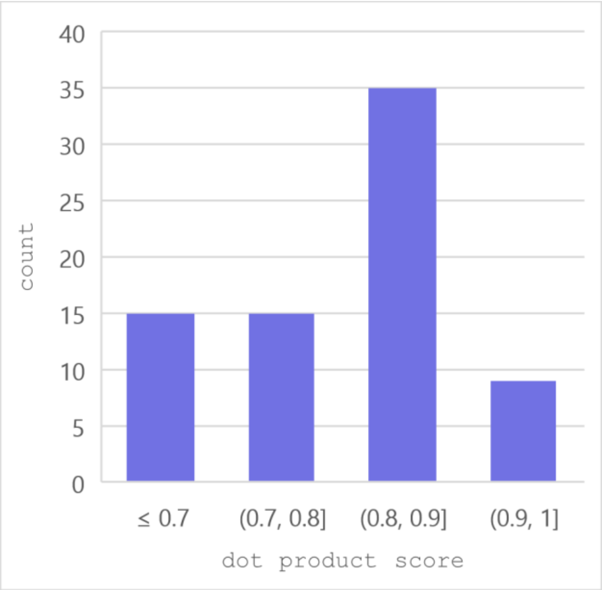

Figure S3. Distribution of the dot product score for DIA data matched against the manually validated spectral library for the Togni labeled data set.

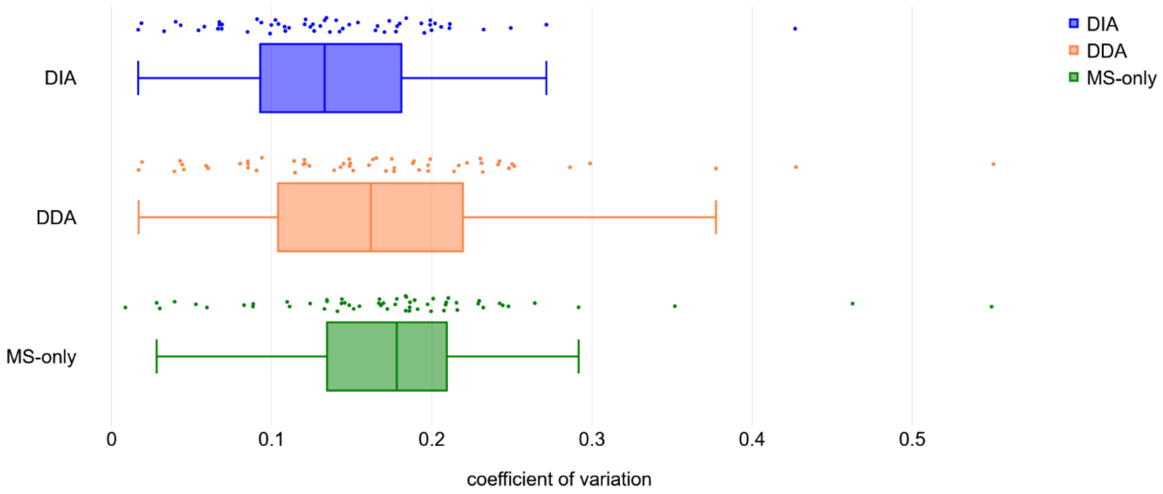

Figure S4. Variability of quantification of the Togni labeled data set. Coefficients of variation were calculated from the quantification of identifications (three replicates) for each acquisition mode.

Table S5. Parameters of CVs for DIA, DDA and MS-only acquisition modes for the Togni labeled data set.

	**DIA**	**DDA**	**MS-only**
**Minimum:**	0.01665	0.01692	0.008634
**Q1:**	0.09288	0.104	0.1346
**Median:**	0.133	0.1619	0.1781
**Q3:**	0.1809	0.2194	0.2093
**Maximum:**	0.4269	0.5507	0.5495
**Mean ($\bar{x}$):**	0.1382	0.1689	0.1781
